# A new oncolytic *Vaccinia virus* augments antitumor immune responses to prevent tumor recurrence and metastasis after surgery

**DOI:** 10.1136/jitc-2019-000415

**Published:** 2020-03-26

**Authors:** Jahangir Ahmed, Louisa S Chard, Ming Yuan, Jiwei Wang, Anwen Howells, Yuenan Li, Haoze Li, Zhongxian Zhang, Shuangshuang Lu, Dongling Gao, Pengju Wang, Yongchao Chu, Chadwan Al Yaghchi, Joel Schwartz, Ghassan Alusi, Nicholas Lemoine, Yaohe Wang

**Affiliations:** 1 Centre for Biomarkers & Biotherapeutics, Barts Cancer Institute, Queen Mary University of London, London, UK; 2 National Centre for International Research in Cell and Gene Therapy, Zhengzhou University, Zhengzhou, Henan, China; 3 University of Illinois at Chicago, Chicago, Illinois, USA

**Keywords:** tumors, surgery, oncology

## Abstract

**Background:**

Local recurrence and remote metastasis are major challenges to overcome in order to improve the survival of patients with cancer after surgery. Oncolytic viruses are a particularly attractive option for prevention of postsurgical disease as they offer a non-toxic treatment option that can directly target residual tumor deposits and beneficially modulate the systemic immune environment that is suppressed post surgery and allows residual disease escape from control. Here, we report that a novel *Vaccinia virus* (VV), VVΔTKΔN1L (with deletion of both thymidine kinase (TK) and N1L genes) armed with interleukin 12 (IL-12), can prolong postoperative survival when used as a neoadjuvant treatment in different murine and hamster surgical models of cancer.

**Methods:**

A tumor-targeted replicating VV with deletion of TK gene and N1L gene (VVΔTKΔN1L) was created. This virus was armed rationally with IL-12. The effect of VVΔTKΔN1L and VVΔTKΔN1L-IL12 on modulation of the tumor microenvironment and induction of tumor-specific immunity as well the feasibility and safety as a neoadjuvant agent for preventing recurrence and metastasis after surgery were assessed in several clinically relevant models.

**Results:**

VVΔTKΔN1L can significantly prolong postoperative survival when used as a neoadjuvant treatment in three different surgery-induced metastatic models of cancer. Efficacy was critically dependent on elevation of circulating natural killer cells that was achieved by virus-induced cytokine production from cells infected with N1L-deleted, but not N1L-intact VV. This effect was further enhanced by arming VVΔTKΔN1L with IL-12, a potent antitumor cytokine. Five daily treatments with VVΔTKΔN1L-IL12 before surgery dramatically improved postsurgical survival. VVΔTKΔN1L armed with human IL-12 completely prevented tumor recurrence in surgical models of head and neck cancer in Syrian hamsters.

**Conclusions:**

These data provide a proof of concept for translation of the regime into clinical trials. VVΔTKΔN1L-IL12 is a promising agent for use as an adjuvant to surgical treatment of solid tumors.

## Introduction

The success of immune checkpoint inhibition at achieving durable remission in a number of cancers has led to a recent evolution in cancer therapy to include immunotherapeutic options as mainstay treatments. Oncolytic viruses (OV) are emerging as a powerful class of immunotherapeutic with the potential to act in synergy with other treatment modalities to improve clinical outcome. Indeed, the recent approval of oncolytic Herpes simplex virus, talimogene laherparepvec (T-VEC), has paved the way in ackowledging OV as validated therapeutics.^1^ OV take advantage of a number of changes in the tumor microenvironment (TME) to facilitate vascular collapse, immunogenic lysis of cancer cells and augmentation of antitumor immune responses, which are often absent in the TME. With the success of T-VEC, we are now moving toward creating the optimal viral vector to maximize the anticancer potency, which can act as stand-alone treatments or complement traditional therapies such as surgical removal of primary tumors.

Surgical excision remains the principle treatment option for solid tumors; however, clinical and experimental data suggest that tumor excision may in fact promote tumor recurrence and metastasis.^2^ The main source of this recurrence is minimal residual disease (MRD) left in situ in microscopic deposits beyond the clearance margins.^3^ A number of mechanisms have been proposed to explain the altered growth of MRD after surgery, including direct dissemination of the tumor cells into the circulatory and lymphatic systems during the procedure, a disinhibition of angiogenesis postoperatively^4^ and the perturbation of the immune system generated by surgical intervention, including marked Th2 polarization and natural killer (NK) cell dysfunction, generating a window of opportunity for tumor immune escape.^2 5^ NK cells are innate immune cytotoxic lymphocytes that have long been implicated in the control of tumor growth and metastasis. An increasing number of clinical and experimental studies directly implicate postsurgical NK dysfunction in metastatic spread of MRD.^6^ Restoration of NK cell function within this window of opportunity therefore represents an important priority for prevention of postoperative recurrence.

Incorporating OV therapy into traditional surgical regimes is an attractive therapeutic option as replication-competent OVs such as *Vaccinia virus* (VV) may be able to directly target and eliminate remaining tumor deposits. OVs are powerful stimulants of antitumor immunity^7^ that can promote long-term tumor immune surveillance^8^ by activating both NK cells and tumor-specific cytotoxic T lymphocytes (CTL), critical antitumor immune effectors that are additionally dysregulated post surgery.^9^ Indeed, it was recently demonstrated that presurgical administration of pox virus could reverse NK cell suppression in experimental models of breast cancer and melanoma in addition to human patients, but the efficacy was limited.^6^ In addition, VV-based OV therapies have strong potential within the framework of postsurgical immune restoration because in addition to the attributes of OV discussed, we have shown that VV entry into tumor cells is facilitated by vascular endothelial growth factor,^10^ levels of which are elevated following surgical stress.^11^

Given the role of NK cells in containing disease, in particular targeting tumor cell populations that are refractory to adaptive immune control via MHCI suppression, and reducing postoperative morbidity, we sought to rationally redesign our thymidine kinase (TK)-deleted Lister strain VV, Vaccinia Virus Lister 15 (VVL15)^12^ to interrupt naturally evolved viral mechanisms of antiviral NK cell suppression with a view to creating a more powerful vector for primary treatment of cancers and additionally optimized as a neoadjuvant therapy. The VV N1L protein is a 13.8 KDa, non-essential virulence determinant^13^ and plays an important role in immune evasion via inhibition of cellular inflammatory pathways and early innate immune responses against viral infection, in particular NK cell activity.^14^ VVs engineered to lack N1L have previously been shown to be attenuated in mice.^15^ N1L inhibits NF-κB signalling in infected cells and deletion has been demonstrated to elevate NK cell responses to viral infection^14^ and improve generation of immediate and long-term memory CD8 +T cell responses,^16^ both of which could be expected to vastly improve the immunotherapeutic potential of oncolytic VV. Here, we demonstrate that intratumoral (i.t.) delivery of N1L-deleted VVL15 (VVLΔTKΔN1L) can control disease and extend survival in subcutaneous models of pancreatic cancer in a T cell-dependent manner. Additionally, by engaging innate immune responses, VVLΔTKΔN1L can reduce metastatic spread from primary tumors and prolong postoperative survival in more aggressive murine models of cancer via upregulation of inflammatory cytokines and circulating NK cells. Efficacy was further enhanced by localized, OV-mediated delivery of the cytokine interleukin 12 (IL-12), consistently demonstrated as one of the most potent antitumor cytokines.^17^

## Materials and methods

### Study approval

All mouse studies were carried out under the terms of the Home Office Project Licence PPL 70/6030 and subject to Queen Mary University of London ethical review, according to the guidelines for the welfare and use of animals in cancer research. Surgical experiments for efficacy studies of 4T1 and LY2 models as well as all hamster procedures were approved by the Animal Welfare and Research Ethics Committee of Zhengzhou University (Zhengzhou, China). For in vivo experiments, power calculations were carried out to determine required sample sizes using G*Power 3, setting parameters of α=0.1, power=90%, effect size=30%, and group=3. In subcutaneous tumor models, animals were assigned to treatment groups by matching tumor sizes prior to treatment. Tumor growth was measured using electronic callipers until tumors measured 1.4 cm (mice) or 1.8 cm (hamster) in diameter or ulcerated, at which point the animals were sacrificed. Tumor growth curves were terminated on the death of the first animal in each group, but group survival was monitored until the experimental endpoint.

### Cell lines

CT26 (metastatic colon adenocarcinoma, BALB/c), CMT93 (rectal adenocarcinoma, C57BL/6), Lewis lung carcinoma (LLC, metastatic lung squamous cell carcinoma, C57BL/6) and B16-F10 (metastatic melanoma, C57BL/6) were obtained from CRUK, Clare Hall, Herts, UK. SCCVII is a murine oral cavity squamous carcinoma cell line (C3H/HeN strain) and was a kind gift of Dr Osam Mazda (Department of Microbiology, Kyoto Prefectural University of Medicine, Kyoto City, Japan). DT6606, a pancreatic ductal adenocarcinoma (PDAC) cell line, derived from LSL-KrasG12D/+; Pdx-1-Cre mice which was a kind gift of Professor David Tuveson (CRUK, Cambridge Research Institute, Cambridge, UK). CV1 (African monkey kidney) cells were obtained from ATCC. LY-2 cell line was isolated from lymph node metastasis after inoculation with PAM212 squamous cell carcinoma cells. They were kindly provided by Dr Carter Van Waes (National Institute of Health, Bethesda, MD, USA). HCPC1 cell line was established from an epidermoid carcinoma of the Syrian golden hamster cheek and was generously provided by Professor Joel Schwartz of University of Illinois.

### Viruses

VVΔTKΔN1L was created using the TK-deleted VVL15 platform.^18^ The N1L right arm was amplified from viral DNA using the primers RA_F: 5′ AAGCTTACGCGTATCTAATAAGTAGAGTCCTCATGCT where HindIII and MluI restriction sites are underlined and RA-R: 5′ GGATCCCGGAAGGTAGTAGCATGGA where the BamHI restriction site is underlined. This fragment was cloned into the multiple cloning region of pUC19 (NEB) via HindIII and BamHI. A red fluorescent protein (RFP) vector with H5 promoter^19 20^ was constructed in pGEM-T (Promega) by amplification of RFP from pCMVdsRedExpress2 (Clonetech Labs) using the primers H5RFP_F: 5′ CCGCGG**AAAAATTGAAAATAAATACAAAGGTTCTTGAGGGTTGTGTTAAATTGAAAGCGAGAAATAATCATAAATA**GCTACCGGACTCAGATCCAC where the SacII restriction site is underlined and the H5 promoter shown in bold. H5RFP_R: 5′ ACGCGTATTTAAATAAGCTT**TATTTATGATTATTTCTCGCTTTCAATTTAACACAACCCTCAAGAACCTTTGTATTTATTTTCAATTTTT**CGCCTTAAGATACATTGATG where HindIII, SwaI and MluI restriction sites are underlined and a second H5 promoter is shown in bold to create a subclone H5-RFP-H5. The left arm of N1L was amplified from viral DNA and subcloned into pGEM-T using the primers LA_F: 5′ AAGCTTGTCCTATCGTAGGCGATAGA where the HindII restriction site is underlined and the reverse primer LA-R: 5′ GTCGAC**TATTTATGATTATTTCTCGCTTTCAATTTAACACAACCCTCAA GAACCTTTGTATTTATTTTCAATTTTT**GATCTAATGATTGATCTATATGGTG where SalI is underlined and a third H5 promoter is shown in bold to create the subclone Left Arm-H5. H5-RFP-H5 was released using SacII and MluI and Left Arm-H5 was released using SalI and HindII. The pUC19 right arm construct was digested with HindII and MluI and the two inserts cloned into this vector to create an N1L shuttle vector; N1L Left Arm-H5-H5-RFP-H5-Right Arm. Virus recombination and production were carried out as described previously.^8 20^ Murine and human IL-12 (for use in mouse and hamster models, respectively) were amplified from pUNO1-mIL12 and pUNO1-hIL12 (Invivogen) using the primers mIL12_F: 5′ AAGCTTATGTGTCCTCAGAAGCTAACCATCTC where HindIII is underlined and mIL12_R: 5′ ATTTAAATCCATACCACATTTGTAGAGG where SwaI is underlined or hIL12_F: 5′ AAGCTTATGTGTCACCAGCAGTTGG and hIL12_R: 5′ ATTTAAACCATACCACATTTGTAGAGG. These fragments were inserted into the HindII/SwaI-digested H5-RFP-H5 subclone prior to creation of the entire shuttle vector to create N1L Left Arm-H5-H5-RFP-H5-m/hIL-21-Right Arm.

### Viral replication and cell cytotoxicity assays

Cell lines were infected with virus at a multiplicity of infection (MOI) of 1 plaque forming units (PFU)/cell. Cells and supernatant were collected at 24, 48 and 72 hours post infection. The median tissue culture infective dose (TCID50) for each sample was calculated as previously described.^8^ The cytotoxicity of the viruses in each cell line was assessed 6 days following infection using an MTS cell proliferation assay kit (Promega), according to the manufacturer’s instructions.

### ELISA

Cytokines/chemokines levels were detected by ELISAs in accordance with the manufacturer’s instructions. Granulocyte-macrophage colony-stimulating factor (GMCSF), IL1α, IL1β, mIL12, hIL12 (Biolegend); granulocyte colony-stimulating factor (GCSF), IL18, keratinocyte chemoattractant (KC; CXCL1) (R&D Systems); macrophage inflammatory protein (MIP)-1α (eBioscience). Murine inflammatory cytokines/chemokines in homogenized tumor tissue were obtained using Qiagen Multi-analyte ELISArray kits.

### Immunophenotyping cells

Single-cell suspensions of tumor or spleen and virally infected antigen-presenting cells (APCs) and macrophages were prepared in fluorescence-activated cell sorting (FACS) buffer (FB; phosphate-buffered saline (PBS) containing 1% heat-inactivated bovine calf serum (BCS)). All fluorophore-conjugated antibodies used at 1:200 dilution and supplied by eBioscience. Cells were blocked with anti-CD16/32 prior to incubation with flurophore-conjugated antibody for 30 min. Cells were fixed in 2% formalin and analyzed using an LSRFortessa multichannel flow cytometer (Beckton Dickinson (BD) Biosciences). Raw data were analyzed using FloJo v10 (FloJo, LLC). Live cells were gated and from these, CD45+ cells selected. Of these, CD8+ T cells (CD3+CD8+), effector CD8+ T cells (CD3+CD8+CD44hiCD62Llo), CD4+ T cells (CD3+CD4+), NK cells (CD3−CD49b+), neutrophils (Cd11b+Gr1+) and macrophages (CD11b+F4/80+) were identified.

### In vivo studies

All in vivo studies were carried out under the terms of the Home Office Project Licence PPL 70/6030 and subject to Queen Mary University of London ethical review, according to the guidelines for the welfare and use of animals in cancer research. 1×10^6^ LLC or LY2 cells or 3×10^6^ DT6606 cells were injected subcutaneously into the right flank of 6-week-old C57/BL6 mice. 1×10^5^ 4T1 cells were injected into the mammary fat pads of female BALB/c mice to establish orthotopic tumors. 2×10^6^ CT26 cells were injected subcutaneously into the right flank of 6-week-old Balb/C mice. 2×10^6^ SCCVII cells were injected subcutaneously into the right flank of 6-week-old C3H/HeN mice. 1×10^7^ HCPC1 cells were injected subcutaneously into the right flank of 4-week-old Syrian hamsters. When tumors were palpable, they were stratified into treatment groups and received i.t. injections of 1×10^8^ PFU of virus or vehicle buffer in a total volume of 50 µL. Tumor growth was measured using electronic callipers until tumors reached a volume of 1.44 cm^3^ at which point the animals were sacrified, and the area calculated according to the following formula:

$$TumorVolume=\frac{\pi w^{2}l}{6}$$

where w is width and l is length.

Tumor growth curves were terminated on the death of the first animal in each group, but group survival was monitored until the experimental endpoint and Kaplan-Meier survival plots generated.

In the neoadjuvant surgical models, tumors were excised under continuous isofluorane and nitrous oxide via nose cone and wounds closed with interrupted 4–0 monocryl absorbable sutures (Ethicon, Livingston, UK). To capture regrowth from microscopic as opposed to macroscopic residual disease, any tumor that clinically regrew within a week of the operation were excluded. Mice were followed up via twice-weekly weight measurements and assessment of general well-being until visibly unwell or a reduction in a maximum of 20% of body weight was noted.

### Immune cell depletion

The VVΔTKΔN1L treatment arm of the LLC surgical neoadjuvant experiment was repeated after depletion of NK, CD4+ or CD8+ cells. One day prior to commencement of i.t. viral treatment, 200 µg of rat anti-CD4 IgG (clone GK1.5), anti-CD8 IgG (clone TIB210), anti-NK IgG (clone PK136) or control IgG in 200 µL PBS was injected intraperitoneally. Injections were continued twice weekly for the duration of the experiment. Depletion was verified using flow cytometric assessment of splenocytes 48 hours post administration.

### Preparation of tumor tissue

Subcutaneous tumors were diced and incubated in collagenase D (1 mg/mL; Roche) and DNAse I (0.1 mg/mL; Roche) in PBS, at 37°C for 2 hours. Homogenates were filtered through 70 µm cell strainers and resuspended in FB.

For cytokine screening, harvested tumors were homogenized in a 10-fold dilution (w/v) of tissue extraction buffer (prepared by dissolving one complete ULTRA minitablet (Roche) into 10 mL of 50 mM Tris–HCl, pH 7.4). Following high-speed centrifugation, the total protein concentration (g/mL) of supernatant samples was obtained using a NanoDrop 1000 Spectrophotometer and subsequently used to normalize multianalyte cytokine/chemokine values.

### Preparation of splenocytes

Harvested spleens were flushed through 70 µm BD Falcon cell strainers with complete T-cell media (RPMI medium 1640 (Sigma Aldrich), 10% FCS, 1% streptomycin/penicillin, 1% sodium pyruvate and 1% non-essential amino acids (Gibco) and 0.1% β-mercaptoethanol). Red blood cells (RBCs) were lysed using RBC lysis buffer (Sigma-Aldrich) and resuspended in complete T-cell medium.

### Preparation of blood leukocytes

Blood was extracted via hepatic vein cannulation and samples were added to a heparinized microcentrifuge tube, resuspended in 1 mL of RBC lysis buffer and filtered through 70 µm cell strainers. Single cells were resuspended in FB.

### Macrophage and dendritic cell (DC) culture

Red marrow was flushed out from femurs and tibias of C57Bl/6 mice using RPMI medium. Cells were homogenized with a 23G needle and syringe, filtered through 70 µm cell strainers and resuspended in RBC lysis buffer followed by resuspension in complete T cell medium containing either M-CSF or GMCSF (Roche) at 30 ng/mL to selectively enrich and grow macrophages or DCs, respectively. At day 7, adherent macrophages were washed with PBS, gently scraped from the plates and resuspended in growth factor-free complete medium. Non-adherent DCs were used at day 7. Purity was confirmed by staining cells with fluorophore-labelled antibodies specifying DCs (CD11c+MHCII+) and monocyte (CD11b+F4/80+) populations. Enriched populations were validated using FACS and infected with the indicted viruses for 24 hours at an MOI of 1 PFU/cell. Infection was confirmed visually by fluorescent microscope observation of virally encoded RFP expression.

### Assessment of lung and lymph node metastases

Flank LLC tumor grafts were monitored via calliper measurement until a treatment group reached the sacrificial end point. All animals were euthanized at the same time; lungs harvested and any gross tumor deposits noted. Lung lobes were separated, fixed in 4% formaldehyde, paraffin embedded and cut into 4 µm sections with a Leica EG1160 microtome (Leica Microsystems UK, Milton Keynes, UK). They were subsequently stained with H&E. For each lobe, slices were also performed above and below the largest cross section. All three sections were scrutinized for tumor deposits via light microscopy by an experienced pathologist who was blinded to treatment groups.

### Statistical analysis

Graph Pad Prism 6 was used for comparative statistical analysis. Dual condition comparisons or normally distributed data were made using the unpaired Student’s t-test. For more than one condition, one or two-way analysis of variances (ANOVAs), respectively, were performed, with post-hoc Tukey tests to compare treatment pairs. Where data were categorical, specific treatment pairs were compared with Fisher’s exact tests following subclassification into multiple 2×2 tables. Survival data were represented by Kaplan-Meier plots with log rank analyses to delineate whether any differences between specific treatment pairs were statistically significant.

## Results

### VVΔTKΔN1L is an effective primary antitumor therapeutic in murine models of pancreatic cancer

**Figure 1:**
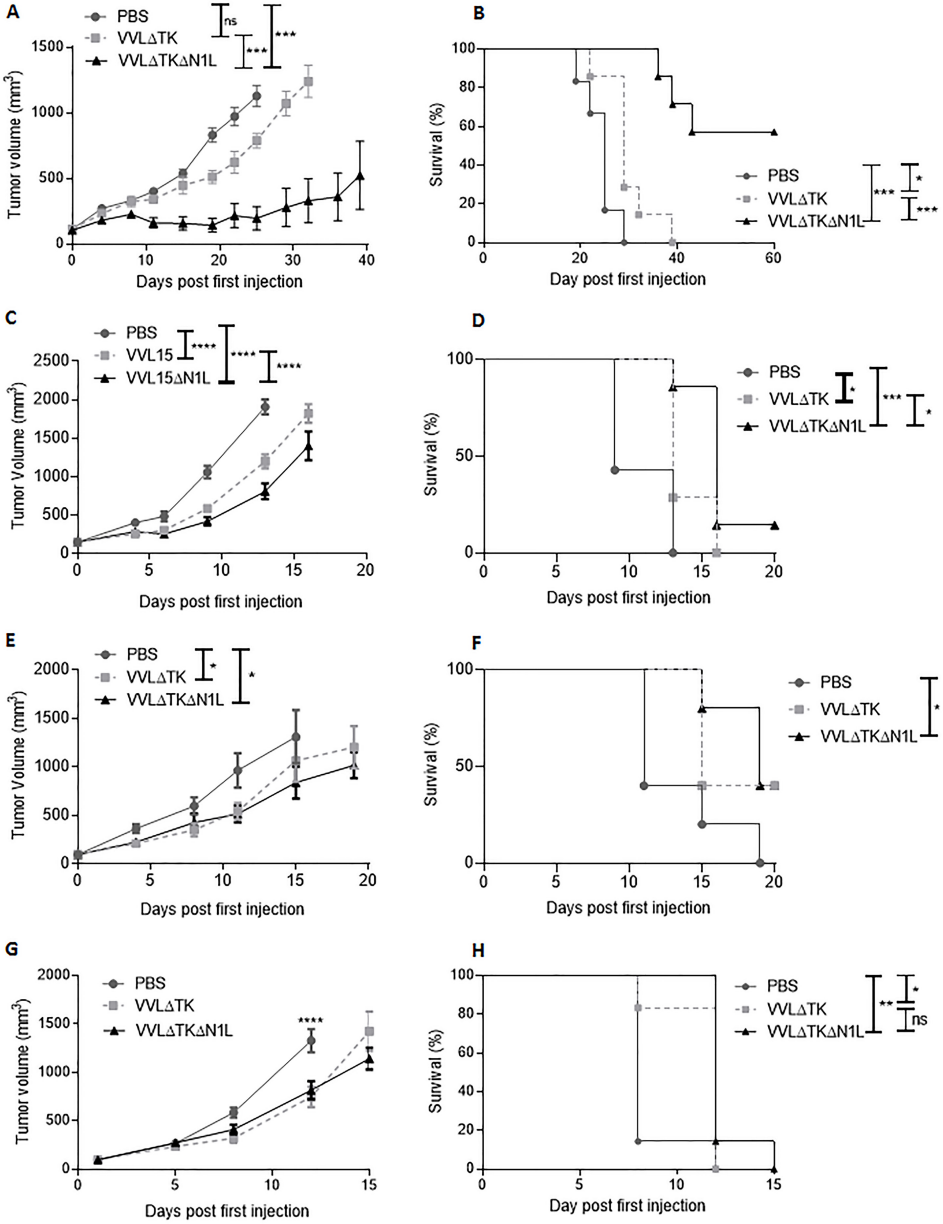
VVΔTKΔN1L demonstrates antitumor efficacy in vivo. (A) and (B) DT6606 pancreatic tumors, (C) and (D) CT26 colorectal tumors, (E) and (F) SCCVII oral cavity squamous carcinoma and (G) and (H) Lewis lung carcinoma were established in the flanks of immune-competent mice (n=5–7/group). Once palpable, mice were injected intratumorally daily for 5 days with 1×10^8^ PFU VVΔTK, VVΔTKΔN1L or PBS. (A), (C), (E), and (G) Tumor growth was monitored and a two-way analysis of variance with post hoc Tukey tests used to compare significance at all time points. Significance at (A) day 25 or (B)–(G) day 20 is shown. (B), (D), (F), and (H) Kaplan-Meier survival analysis with Log rank (Mantel-Cox) tests was used to assess survival. In all cases, the mean±SEM is shown. *p<0.05; **p<0.01; ***p<0.001; ****p<0.0001.

The TK-deleted VV, VVL15, was manipulated as described in the methods to interrupt the N1L region, replacing it with an RFP marker under control of the endogenous H5 promoter, creating VVΔTKΔN1L. The deletion of N1L did not negate cytotoxicity compared with VVL15 (VVΔTK) in all murine and human tumor cell lines examined (online supplementary figure S1A) and the virus remained replication competent. Replication was modestly, but significantly attenuated in many cell lines, which was unsurprising given that viral replication has been inversely correlated to the extent to which it has been manipulated (online supplementary figure S1B). In vivo, VVΔTKΔN1L was able to exert significantly more control over murine pancreatic DT6606 subcutaneous tumor growth compared with VVΔTK, which translated into substantially improved survival in this model (figure 1A, B). This effect was also evidence, although far less pronounces in a CT26 model of colorectal cancer (figure 1C, D) and more aggressive SCCVII squamous cell carcinoma (figure 1E, F) and LLC metastatic lung cancer (figure 1G, H) flank models, in which both viruses were only able to exert limited control over tumor growth, although VVΔTKΔN1L was able to extend survival significantly compared with PBS treatment in each model.

10.1136/jitc-2019-000415.supp1 Supplementary data

10.1136/jitc-2019-000415.supp6 Supplementary data

### VVΔTKΔN1L induces adaptive antitumor immune responses to mediate efficacy in murine models of pancreatic cancer

**Figure 2:**
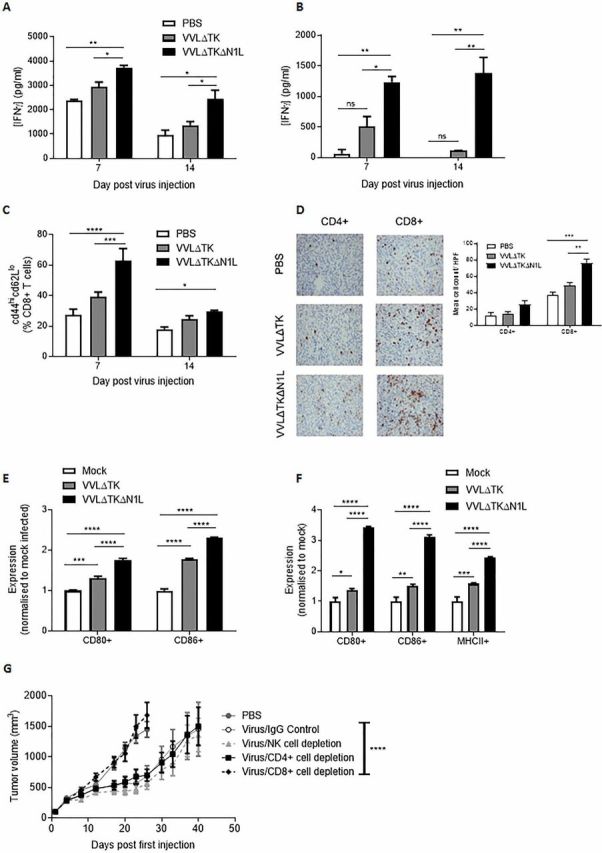
VVΔTKΔN1L induces adaptive immune responses against pancreatic cancer in vivo. DT6606 tumors were established in the flanks of immune-competent C57/Bl6 mice (n=3–4/group). Once palpable, mice were injected intratumorally (i.t) once with 1×10^8^ PFU VVΔTK, VVΔTKΔN1L or PBS. Splenocytes were collected 7 or 14 days after treatment and analyzed. (A) Splenocytes were analyzed ex vivo for response to growth-arrested DT6606 cells using coculture for 72 hours followed by interferon γ (IFNγ) ELISA. (B) Splenocytes were analyzed ex vivo for response to mesothelin peptide using IFNγ ELISA after a 72-hour stimulation. (C) Splenocytes were collected and analyzed using FACS for expression of CD45, CD3, CD8, CD44 and CD62L. Percentage CD44^hi^CD62L^lo^ cells (of live, CD45+/CD3+/CD8+ cells) are shown. (D) Tumors were collected 14 days post treatment and stained for CD4+ or CD8+ cells. Representative immunohistochemistry (IHC) images are shown (original magnification x200) and cells/higher power field (HPF) shown graphically after 15 HPFs were counted. Of note, CD4+ staining cannot exclude the presence of TReg cells within the tumor. (E) and (F) Dendritic cells (DCs) and macrophages were matured from bone marrow precursors taken from C57/Bl6 mice using granulocyte-macrophage colony-stimulating factor and macrophage colony-stimulating factor, respectively. Enriched populations were validated using FACS and infected with the indicted viruses for 24 hours at an MOI of 1 PFU/cell (n=3/group). (E) Expression of CD80 and CD86 markers on enriched DCs was examined after in vitro infection using FACS and normalized to expression levels on mock (PBS)-infected cells. (F) Expression of CD80, CD86 and major histocompatibility complex II (MHCII) markers on enriched macrophages was examined after infection using FACS and normalized to expression levels on mock (PBS)-infected cells. In all cases, one-way analysis of variance (ANOVA) with post hoc Tukey tests was used to assess significance at each time point. (G) Cell depleting or IgG control antibodies were commenced intraperitoneally in mice bearing DT6606 flank tumors a day prior to the first i.t treatment of virus (n=7/group). Mice were treated as above and tumor growth monitored. A two-way ANOVA with post hoc Tukey tests was used to assess significance. In all cases, the mean±SEM is shown. *p<0.05; **p<0.01; ***p<0.001; ****p<0.0001.

Given the ability of VVΔTKΔN1L to control DT6606 murine pancreatic tumors, we investigated the functional mechanisms responsible for extended survival in this model. Ex vivo restimulation of splenocytes with growth-arrested tumor cells demonstrated a significant enhancement of tumor-specific IFNγ production up to 2 weeks after mice were treated with a single dose of VVΔTKΔN1L (figure 2A), an effect also evident when splenocytes were restimulated ex vivo with a peptide representing the mesothelin epitope, overexpressed in pancreatic cancer (figure 2B).

Cytotoxic T cell assays confirmed the superior ability of splenocytes from VVΔTKΔN1L-treated animals to specifically lyse target tumor cells, but not unrelated cells, ex vivo (online supplementary figure S2A). FACS analysis of splenocytes at days 7 and 14 post injection indicated that VVΔTKΔN1L treatment enhanced effector CD8+ populations (figure 2C) and increased the number of CD8+ and CD4+ T cells infiltrating into tumors (figure 2D), demonstrating an effective impact on generation of antitumor adaptive immune responses in vivo. In vitro, VVL recombinants were able to enhance activation of both bone marrow-derived DCs (figure 2E) and macrophages (figure 2F) and VVΔTKΔN1L treatment resulted in a shift of the macrophage pool into professional APCs as evidenced by the significant upregulation of MHCII 24 hours post infection, suggesting a phenotypic switch of macrophages to M1-polarized antitumor populations. To further confirm the requirement for adaptive immunity to mediate the observed therapeutic effect, immune subset depletion assays were carried out that demonstrated primary antitumor efficacy could be maintained when CD4+ T and NK cells were depleted, but CD8+ T cells were absolutely required to deliver a therapeutic effect after treatment (figure 2G). These data suggest that the therapeutic efficacy of VVΔTKΔN1L is conferred by enhanced APC and T cell activation and intratumoral infiltration in the murine pancreatic model.

10.1136/jitc-2019-000415.supp2 Supplementary data

### VVΔTKΔN1L decreases metastasis to extend survival in an aggressive murine model of metastatic lung cancer

**Figure 3:**
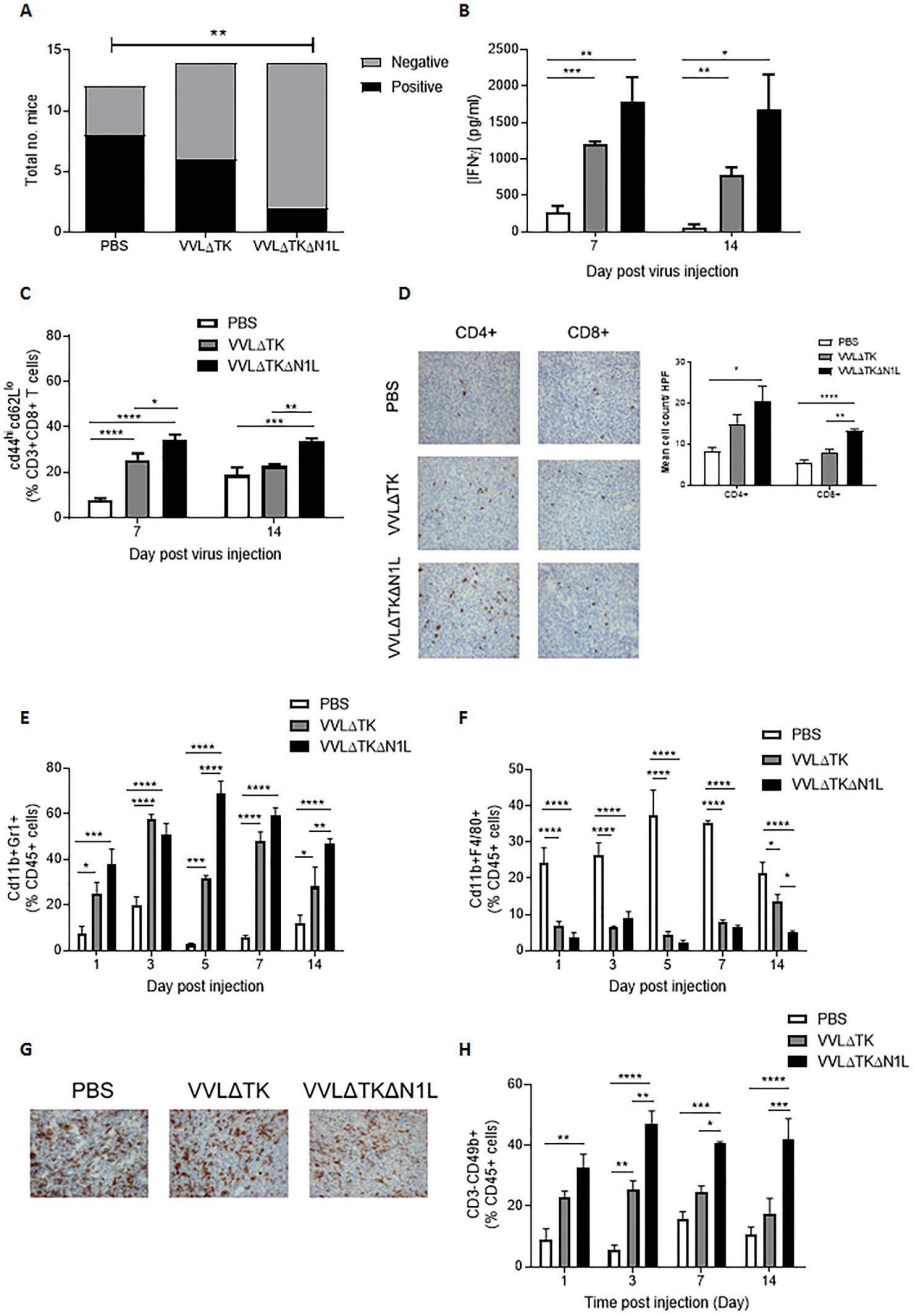
VVΔTKΔN1L induces adaptive and innate immunity against lung cancer in vivo. Lewis lung carcinoma (LLC) tumors were established in the flanks of immune-competent C57/Bl6 mice. (A) Once palpable, mice were injected intratumorally (i.t) daily for 5 days with 1×10^8^ PFU VVΔTK, VVΔTKΔN1L or PBS (n=14/group). Mice were sacrificed at day 15, lungs H&E stained and analyzed for the presence of metastasis by a pathologist blinded to treatment groups. Lungs were scored as negative or positive and a Fishers exact test used to determine significance. (B)–(H) Once palpable, mice were injected i.t once with 1×10^8^ PFU VVΔTK, VVΔTKΔN1L or PBS (n=3–4/group). (B) Splenocytes were analyzed ex vivo for response to growth-arrested LLC cells using coculture for 72 hours followed by interferon γ (IFNγ) ELISA. (C) Splenocytes were collected and analyzed using FACS for expression of CD45, CD3, CD8, CD44 and CD62L. Percentage CD44^hi^CD62L^lo^ cells (of live, CD45+/CD3+/CD8+ cells) are shown. (D) Tumors were collected 14 days post treatment and stained for CD4+ or CD8+ cells. Representative IHC images are shown (magnification x200) and cells/HPF shown graphically after 15 HPFs were counted. Of note, CD4+ staining cannot exclude the presence of TReg cells within the tumor. (E) FACS analysis was used to assess CD11b+Gr1+ neutrophils in tumors at the indicated time points. (F) FACS analysis was used to assess CD11b+F4/80+ macrophages in the tumors at the indicated time points. (G) Fourteen days post infection tumor sections were stained with F4/80 to assess macrophage infiltration. Representative images are shown. Magnification x200. (H) FACS analysis was used to assess CD3−CD49b+ natural killer cells in blood at the indicated time points. In all cases, one-way analysis of variance with post hoc Tukey tests was used to assess significance at each time point. In all cases, the mean±SEM is shown. *p<0.05; **p<0.01; ***p<0.001; ****p<0.0001.

VVΔTKΔN1L was not effective at controlling primary tumor burden in more aggressive tumor models, but it was noted that in the murine lung cancer model survival was significantly, but modestly, extended after treatment compared with treatment using VVΔTK. Analysis of lung metastasis in this model suggested that this survival effect was associated with superior control of metastatic spread of the primary tumor (online supplementary figure 3A, S2B). Ex vivo restimulation of splenocytes with growth-arrested tumor cells again demonstrated a significant enhancement of tumor-specific IFNγ production up to 2 weeks after mice were treated with a single dose of VVΔTKΔN1L (figure 3B), increased activation and cytotoxicity of CD8+ T cells (figure 3C; online supplementary figure S2C) and increased infiltration of CD4+ and CD8+ T cells into the tumor (figure 3D). However, the extent to which VVΔTKΔN1L was able to activate and elevate T cells was low in comparison to those seen using the DT6606 pancreatic cancer model. The LLC model of lung cancer is an extremely aggressive subcutaneous model and although VVΔTKΔN1L was able to enhance T cell infiltration into the tumor and generate effective antitumor effector T cell responses, the time needed to generate these responses and the comparatively weak responses generated is likely to be too weak and slow to prevent demise of the animal by tumor dissemination.

10.1136/jitc-2019-000415.supp3 Supplementary data

As VVΔTKΔN1L was able to limit metastasis in this model to a significantly greater extent than VVΔTK treatment, we investigated early induction of innate immune responses as a potential explanation for metastatic control. Following a single i.t. dose of virus into LLC tumor-bearing mice, both viruses were able to enhance neutrophil infiltration into the tumor, with VVΔTKΔN1L having a superior effect compared with VVΔTK (figure 3E). Conversely, macrophage populations within the tumor were significantly reduced by viral treatment (figure 3F, G). These results were mirrored in similar experiments investigating neutrophil and macrophage accumulation in DT6606 tumors (online supplementary figure S2D, E). i.t. NK cells were undetectable; however, VVΔTKΔN1L treatment was able to induce a significant increase in circulating NK cells, critical for control of tumor dissemination from the primary tumor, at early time points after injection into LLC tumors (figure 3H). These results suggest that in aggressive tumor models, the adaptive immune environment programmed by VVΔTKΔN1L is insufficient to control the rapidly developing primary tumor, but the innate immunity changes conferred by treatment are able to reduce metastatic spread from the primary tumor, extending mortality in treated animals.

### VVΔTKΔN1L can perturb the biochemical microenvironment of the tumor in an aggressive murine lung cancer model

**Figure 4:**
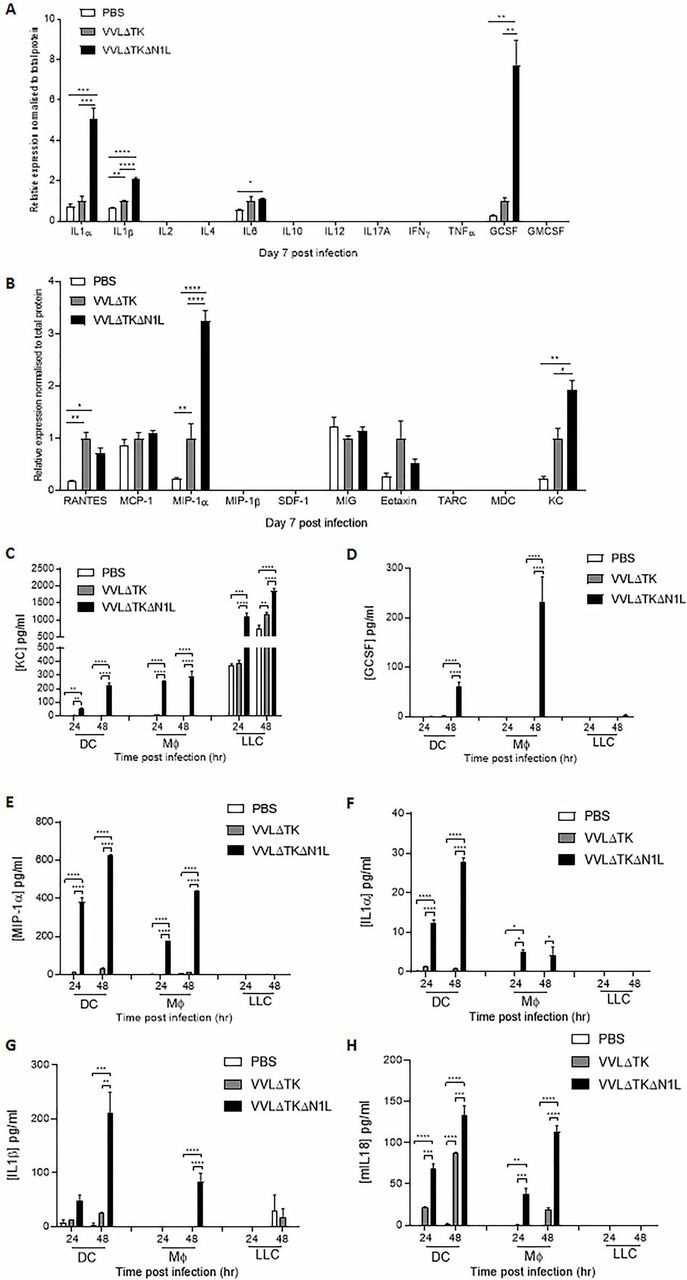
VVΔTKΔN1L alters antigen-presenting cell secretion of immune modulators. (A) and (B) Lewis lung carcinoma (LLC) tumors were established in the flanks of immune-competent C57/Bl6 mice (n=3–4/group). Once palpable, mice were injected intratumorally once with 1×10^8^ PFU VVΔTK, VVΔTKΔN1L or PBS. One-way analysis of variance (ANOVA) with post hoc Tukey tests was used to assess significance. (A) At 7 days post infection, tumors were harvested and homogenized and their supernatant analyzed for the presence of 12 common inflammatory cytokines by multianalyte ELISA. Relative expression was normalized to total protein. (B) At 7 days post infection, tumors were harvested and homogenized and their supernatant analyzed for the presence of 12 common inflammatory chemokines by multianalyte ELISA. Relative expression was normalized to total protein. (C)–(H) Dendritic cell (DC) and macrophage populations were matured from bone marrow as previously and infected with virus at an MOI of 1 PFU/cell for 24 and 48 hours. LLC cells were infected and analyzed in parallel. (C) Supernatants were analyzed for the presence of keratinocyte chemoattractant (KC) using ELISA. (D) Supernatants were analyzed for the presence of granulocyte colony-stimulating factor (GCSF) using ELISA. (E) Supernatants were analyzed for the presence of macrophage inflammatory protein (MIP)-1α using ELISA. (F) Supernatants were analyzed for the presence of interleukin (IL)-1α using ELISA. (G) Supernatants were analyzed for the presence of IL-1β using ELISA. (H) Supernatants were analyzed for the presence of IL-18 using ELISA. Two-way ANOVA with post hoc Tukey tests was used to assess significance. In all cases, the mean±SEM is shown. *p<0.05; **p<0.01; ***p<0.001; ****p<0.0001. GMCSF, granulocyte-macrophage colony-stimulating factor; IFNγ, interferon γ. RANTES; regulated on activation, normal T cell expressed and secreted, TNFa; tumor necrosis factor a, MCP-1; Monocyte Chemoattractant Protein-1, SDF-1; stromal cell-derived factor 1, MIG; monokine induced by gamma interferon, TARC; Thymus- And Activation-Regulated Chemokine, MDC; Macrophage-derived Chemokine.

To delineate how VVΔTKΔN1L was able to effect early innate cellular changes, syngeneic LLC flank tumors were collected after one i.t. dose of virus and screened for common inflammatory cytokines and chemokines via multianalyte ELISAs. IL-α, IL1-β and GCSF cytokines were consistently significantly elevated after VVΔTKΔN1L treatment (figure 4A; online supplementary figure S3A-C), likewise for the chemokines MIP-1α and KC (figure 4B; online supplementary figure S3D-E). Interestingly, IL-12, a major DC-secreted mediator of NK effector function, was not detected in tumors infected with either virus.

Investigations into the sources of elevated immune mediators revealed that the murine chemokine KC (CXCL1), a functional homologue of human IL-8 important for neutrophil recruitment, was produced at much higher levels following infection of LLC tumor cells in comparison to DCs or monocytes (figure 4C). Infection with VVΔTKΔN1L enhanced production of KC in comparison to VVΔTK across all cell lines, although uninfected LLC tumor cells produced significant basal levels of KC. GCSF aids the recruitment and maturation of neutrophils and other myeloid cells and its production from infected DCs and monocytes occurred almost universally following VVΔTKΔN1L infection (figure 4D). MIP1α, a chemokine facilitating the recruitment and activation of myeloid-derived cells, NK cell migration and the generation of a CD8+ cytotoxic T lymphocyte (CTL) memory responses^21^ were also secreted from infected DCs and macrophages and VVΔTKΔN1L infection markedly increased output from both these cell populations in vitro (figure 4E). The classic acute phase reactant cytokines, IL1-α and IL1-β, play a major role in the recruitment and activation of macrophages and neutrophils as well as lymphocytes. Further, IL1-β may help drive a Th-1 type antigen-specific CTL response.^22^ Both these cytokines were secreted from DCs and macrophages almost exclusively in response to VVΔTKΔN1L (figure 4F, G). In addition, VVΔTKΔN1L was also able to significantly enhance IL-18 secretion from DCs and macrophages in vitro (figure 4H). IL-I β and IL-18 act as surrogate markers for intracellular activation of inflammasome platforms, suggesting that the VV N1L protein may additionally downregulate inflammasome signaling. Thus, it appears that by releasing suppression on the NFκB pathway, VVΔTKΔN1L can dramatically alter the levels of inflammatory mediators expressed within the TME, the major source of which appear to be macrophages and DCs. These changes can explain the profound effects on innate NK and neutrophil populations and also the elevation in T cell responses seen as a result of OV treatment.

### VVΔTKΔN1L can enhance postoperative survival in aggressive metastatic tumor models

**Figure 5:**
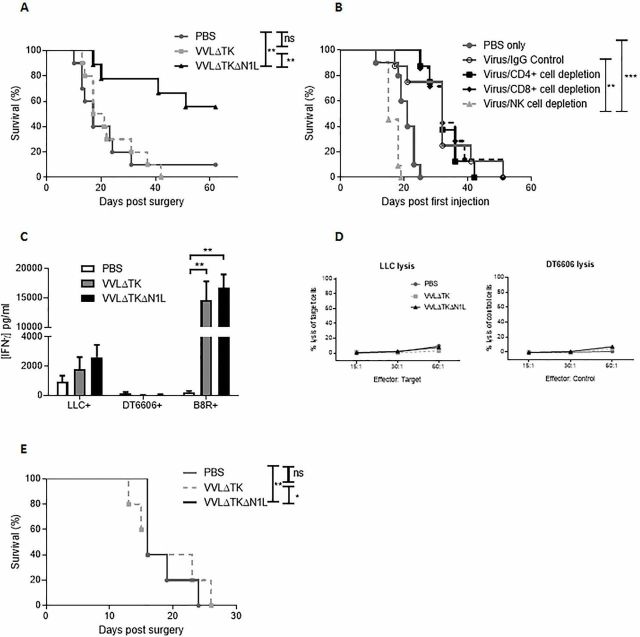
VVΔTKΔN1L is an effective surgical adjuvant in vivo. (A) Lewis lung carcinoma (LLC) tumors were established in the flanks of immune-competent C57/Bl6 mice (n=7–10/group). Once palpable, mice were injected intratumoral (i.t) with 1×10^8^ PFU VVΔTK, VVΔTKΔN1L or PBS daily for 5 days. Tumors were surgically resected 7 days after the final virus dose. Kaplan-Meier survival analysis with log rank (Mantel-Cox) tests was used to assess survival. (B) Cell depleting or IgG control antibodies were commenced intraperitoneally in mice bearing LLC flank tumors a day prior to the first i.t treatment of virus (n=7/group). Mice were treated and resected as in (A) and Kaplan-Meier survival analysis with log rank (Mantel-Cox) tests was used to assess survival. (C) Mice were treated as in (A) and 7 days post resection, splenocytes were analyzed for response to growth-arrested LLC or DT6606 (control) tumor cells ex vivo using interferon γ (IFNγ) ELISA after 72 hours coculture (n=3–4/group). Response of the splenocytes to the *Vaccinia virus* B8R peptide was assessed in parallel. One-way analysis of variance with post hoc Tukey tests was used to assess significance. (D) Splenocytes isolated as in (C) were cocultured with growth-arrested tumor cells for 5 days, pooled and further cultured with 5000 target (LLC) or control (DT6606) tumor cells at 15:1, 30:1 and 60:1 ratios. Non-radioactive Lactate dehydrogenase (LDH) release assays were performed to determine percentage tumor cell lysis. (E) Orthotopic 4T1 breast tumors were established in immune-competent Balb/C mice (n=5/group) and treated with three doses of virus at 1×10^8^ PFU. Five days later, tumors were surgically resected. Kaplan-Meier survival analysis with log rank (Mantel-Cox) tests was used to assess survival. In all cases, the mean±SEM is shown. *p<0.05; **p<0.01; ***p<0.001. NK, natural killer.

LLC tumors avidly infiltrate underlying tissue and readily metastasize to the lungs; thus, surgical excision is likely to leave foci of residual disease, which has been well characterized as an ideal model of MRD postsurgical resection.^23 24^ Given the suppressive effects of the N1L protein on NK populations and the involvement in NK cells in control of tumor dissemination, we next explored the use of VVΔTKΔN1L as a neoadjuvant alongside surgical excision of tumors, a scenario which may better realize the curative potential of OV therapy. Twelve days prior to surgical excision, PBS, VVΔTK or VVΔTKΔN1L were administered i.t. daily for 5 days. There was a significant postoperative survival advantage favoring neoadjuvant i.t. VVΔTKΔN1L treatment (figure 5A). Given the effects of VVΔTKΔN1L on systemic T and NK populations, the VVΔTKΔN1L neoadjuvant therapy regime was repeated in LLC flank tumor-bearing mice in which NK, CD4+ or CD8+ T cells were selectively depleted. The efficacy of VVΔTKΔN1L as a surgical adjuvant was abrogated only when NK cells were depleted (figure 5B), demonstrating that the proliferation and mobilization of NK, but not T cells can delay postsurgical mortality in this model. Indeed, analysis of postsurgical adaptive immune responses demonstrates that the antitumor immunity generated on vaccination using VVΔTKΔN1L was lost after surgery, further demonstrating that NK cells are the critical component of the efficacy of VVΔTKΔN1L at preventing postoperative mortality (figure 5C, D).

To demonstrate broader application of this result, response of orthotopically implanted metastatic 4T1 breast cancer was examined, which again demonstrated a significant prolongation of survival following treatment with VVΔTKΔN1L prior to surgical excision (figure 5E).

### IL-12 can enhance the primary therapeutic and neoadjuvant potential of VVΔTKΔN1L

**Figure 6:**
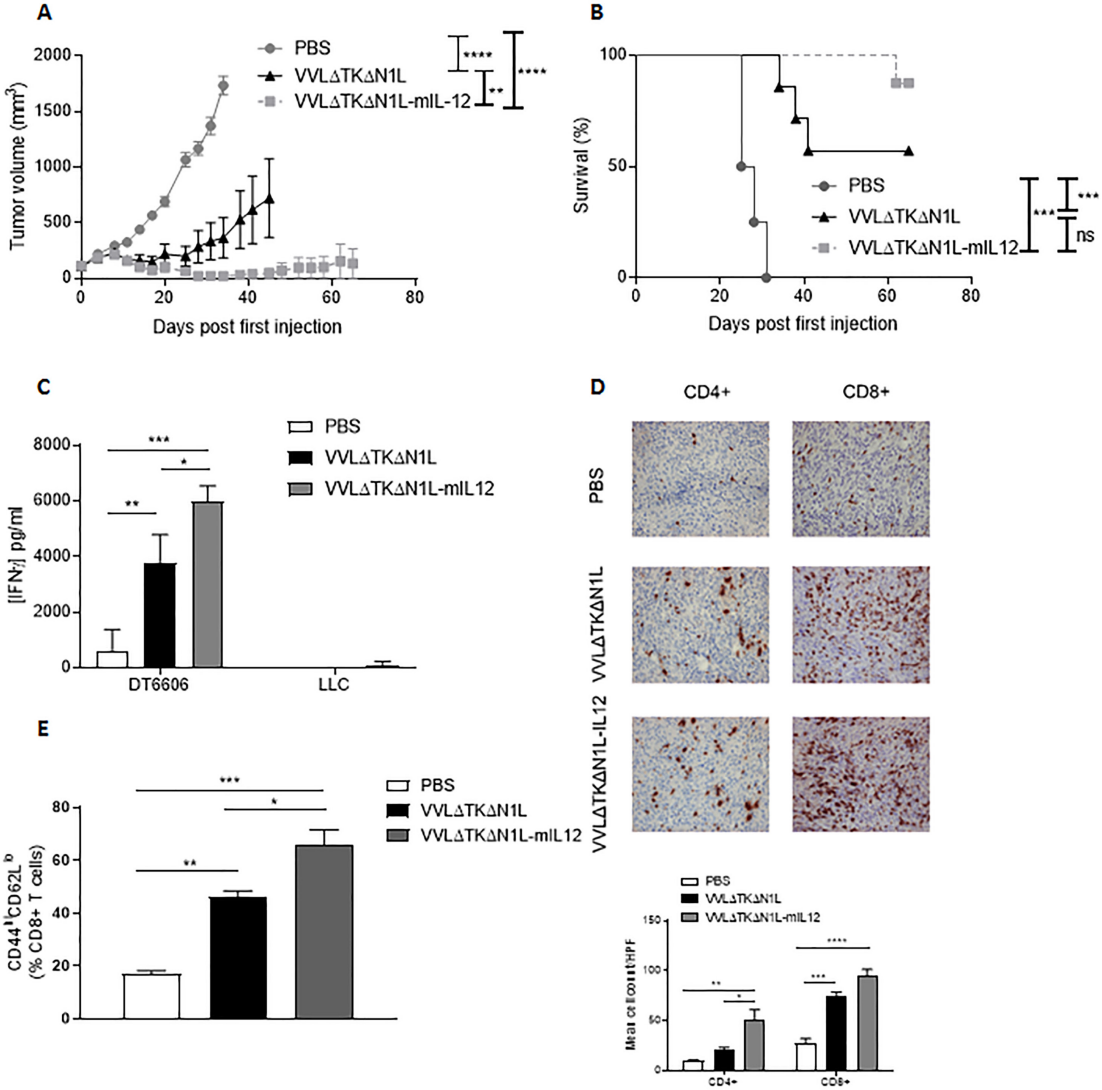
Arming VVΔTKΔN1L with interleukin (IL)-12 improves efficacy as a monotherapeutic agent. (A)–(E) DT6606 tumors were established in the flanks of immune-competent C57/Bl6 mice (n=6–7/group). Once palpable, mice were injected intratumoral with 1×10^8^ PFU VVΔTKΔN1L, VVΔTKΔN1L-mIL12 or PBS daily for 5 days. (A) Tumor growth was monitored and significance at each time point analyzed using two-way analysis of variance (ANOVA) with post hoc Tukey tests. Significance at day 34 is shown. (B) Kaplan-Meier survival analysis with log rank (Mantel-Cox) tests was used to assess survival. (C) DT6606 flank tumors were treated as above (n=3–4/group) and 14 days after the first treatment splenocytes were analyzed for response to growth-arrested Lewis lung carcinoma (LLC) or DT6606 (control) tumor cells ex vivo using interferon γ (IFNγ) ELISA after 72 hours coculture. (D) DT6606 tumors were treated as above and 14 days after the first treatment, tumor sections were immunostained with CD4 or CD8 antibodies (n=3–4/group). Representative images are shown (magnification x200) and a manual cell count pre-HPF (taken from 15 HPFs) depicted graphically. One-way ANOVA with post hoc Tukey tests was used to assess significance. (E) DT6606 tumors were treated as above (n=3–4/group) and 14 days after the first treatment, splenocytes analyzed using FACS for activated CD8+ T cells using CD44 and CD62L (of live, CD45+, CD3+, CD8+ populations). One-way ANOVA with post hoc Tukey tests were used to assess significance. In all cases, the mean±SEM is shown. *p<0.05; **p<0.01; ***p<0.001.

IL-12 is a pleiotropic cytokine with strong antitumor potential and known effects on both adaptive and innate components on the immune system. We reasoned that by arming VVΔTKΔN1L with IL-12, the effects of viral therapy on the innate (NK) and adaptive (T cell) populations could be augmented, creating a powerful therapeutic for use in primary or neoadjuvant settings. Increased IL-12 expression was detected after viral infection of tumor cell lines with VVΔTKΔN1L-mIL12 (expressing murine IL-12) and VVΔTKΔN1L-hIL12 (expressing human IL-12) in vitro and the virus retained the capacity to replicate in and kill tumor cells (online supplementary figure S4). In vivo, treatment of DT6606 subcutaneous pancreatic tumors, which responded well to VVΔTKΔN1L, was dramatically enhanced by the addition of IL-12 to the virus, affecting a 90% cure rate, compared with 60% using the unarmed virus (figure 6A, B).

10.1136/jitc-2019-000415.supp4 Supplementary data

Enhanced efficacy was reflected by improved tumor-specific immunity (figure 6C), increased T cell infiltration into tumors (figure 6D) and increased activation of splenic CD8+ T cells (figure 6E) demonstrating VVΔTKΔN1L-mIl12 as a powerful monotherapeutic agent that can mediate long-term control of tumors by augmentation of adaptive antitumor immune responses.

**Figure 7:**
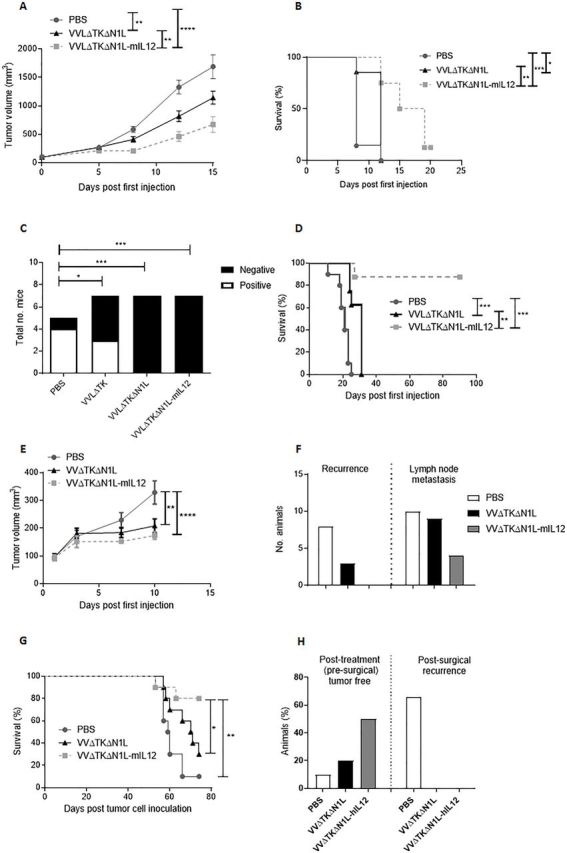
VVΔTKΔN1L-IL12 is an effective surgical adjuvant treatment in vivo. (A) Lewis lung carcinoma (LLC) tumors were established in the flanks of immune-competent C57/Bl6 mice (n=7/group). Once palpable, mice were injected intratumoral (i.t) with 1×10^8^ PFU VVΔTKΔN1L, VVΔTKΔN1L-mIL12 or PBS daily for 5 days (n=8–10/group). (A) Tumor growth was monitored until resection. A two-way analysis of variance (ANOVA) with post hoc Tukey tests was used to assess significance at each time point. (B) Overall survival was monitored using Kaplan-Meier survival analysis with log rank (Mantel-Cox) tests. (C) LLC tumors were treated as above. Tumors were surgically resected 7 days after the final dose of virus. Mice were sacrificed at day 15, H&E stained and analyzed for the presence of metastasis by a pathologist blinded to treatment groups. Lungs were scored as negative or positive and a Fishers exact test used to determine significance. (D) Mice treated in (C) were assessed for long-term survival after treatment and surgical excision of tumors using Kaplan-Meier survival analysis with log rank (Mantel-Cox) tests. (E) LY2 murine head and neck tumors were established in the flanks of immune-competent C57/Bl6 mice (n=10/group). Once palpable, mice were injected i.t with 1×10^8^ PFU VVΔTKΔN1L, VVΔTKΔN1L-mIL12 or PBS daily for 5 days. Tumor growth was monitored until resection 6 days following the last treatment. A two-way ANOVA with post hoc Tukey tests was used to assess significance at each time point. Significance at day 10 is shown. (F) LY2 tumors were treated as above and recurrence at the excision site or lymph node metastasis on sacrifice recorded. (G) LY2 tumors were treated as above and survival post resection monitored using Kaplan-Meier survival analysis with log rank (Mantel-Cox) tests. (H) Hamster HCPC1 tumors were established in the flank of Syrian hamsters. Once palpable, hamsters were injected i.t with 1×10^8^ PFU VVΔTKΔN1L, VVΔTKΔN1L-hIL12 or PBS daily for 5 days (n=10/group). The effect of treatment on the primary tumor is shown. Six days following the last treatment, remaining tumors were surgically excised (PBS n=9; VVΔTKΔN1L n=8; VVΔTKΔN1L-hIL12 n=5) and recurrence monitored. In all cases, the mean±SEM is shown. *p<0.05; **p<0.01; ***p<0.001; ****p<0.0001.

We next assessed the efficacy of VVΔTKΔN1L-mIL12 as a neoadjuvant to surgery using the more aggressive LLC model. Subcutaneous LLC tumors were injected five times daily with VVΔTKΔN1L or VVΔTKΔN1L-mIL12 and the tumor growth monitored. Growth of the primary tumor was controlled significantly better and overall survival improved when the virus was armed with IL-12, demonstrating the value of this addition for presurgical debulking of tumors (figure 7A, B). Encouragingly, when applied as a surgical adjuvant, VVΔTKΔN1L-mIL12 was able to maintain control of lung metastasis (figure 7C) and significantly extend long-term survival of mice (figure 7D).

We next explored the antitumor efficacy of VVΔTKΔN1L-mIL12 in an LY2 murine model of head and neck cancer, representing a model that is intrinsically amenable to neoadjuvant i.t. OV treatment and surgical resection. LY2 models were established subcutaneously in immunocompetent mice and i.t. OV treatment given for 5 days. Six days following the last OV treatment, tumors were excised. Both viruses were able to exert initial control over tumor growth in this model (figure 7E) and OV treatment was able to control both tumor recurrence at the excision site and lymph node metastasis (figure 7F), with VVΔTKΔN1L-mIL12 outperforming VVΔTKΔN1L. Importantly, VVΔTKΔN1L-mIL12 was able to affect a long-term cure in 80% of animals (figure 7G). We have previously shown that i.t. treatment with an IL-12 expressing OV is non-toxic^25^ and this was confirmed in the LY2 model, in which there was no unresolved perturbation of liver and kidney biochemistry after treatment (online supplementary figure S5). VVΔTKΔN1L expressing human IL12 (VVΔTKΔN1L-hIL12), that is fully functional in Syrian hamsters,^25^ was further investigated using a Syrian Hamster subcutaneous model of head and neck cancer. HCPC1 tumors were established and treated as previously prior to surgical excision. These tumors are particularly sensitive to OV treatment and VVΔTKΔN1L was able to affect complete tumor regression prior to surgical excision in 2/10 animals. VVΔTKΔN1L-hIL12 was able to affect presurgical cure in 5/10 animals. Surgical removal of the remaining tumors affected a complete cure, while 6/9 PBS-treated animals demonstrated tumor recurrence by 15 days post surgery (figure 7H).

10.1136/jitc-2019-000415.supp5 Supplementary data

Overall, these results demonstrate that IL-12 augments the activity of VVΔTKΔN1L as a monotherapy for primary tumors by enhancing T cell responses to the tumor. Its potency against aggressive, metastatic tumors is less effective; however, when combined with surgical intervention, it is able to prevent postoperative mortality by preventing recurrence and metastasis that occurs commonly following surgical intervention.

## Discussion

Appropriate activation of the innate immune system is a prerequisite to effective priming of adaptive immune responses against both foreign and self-antigens, including tumor-associated antigen (TAAs).^26^ Viruses are powerful stimulants of the innate immune system and there is an accumulating body of evidence that this effect in its own right may mediate the antitumor effects of some OVs. In a syngeneic colorectal tumor model treated with the Western reserve strain of VV (WRDD), massive tumor necrosis occurred consequent to the influx of neutrophils interrupting tumor vascularity,^27^ while NK cell depletion abrogated tumor clearance following treatment with other OVs.^28^

Animal and clinical studies have demonstrated that surgical manipulation may enhance postoperative metastases.^2 29^ Indeed surgical excision of subcutaneously grown LLC tumor has been shown to enhance the rate of lung metastases via removal of an angiogenesis inhibitor secreted by the primary tumor.^23^ Immune suppression is also emerging as a key factor in postsurgical disease spread and components of the innate immune response, including NK responses, are known to be dampened.^2 6 29^ Surgery-induced immune suppression peaks 3 days post surgery, but the restoration of immune competence may take up to 5 weeks,^2^ generating a significant window of opportunity for tumor dissemination.

At present, there are no FDA-approved perioperative approaches aimed at controlling MRD. Perioperative cytokine therapies using IL-2 (IL-2)^30^ and interferon-α (IFN-α)^31^ have both shown encouraging effects by preventing postoperative NK suppression; however, these therapies have not been developed further due to substantial dose-limiting toxicities.^32^ Therefore, development of neoadjuvant therapeutic regimes for prevention of recurrence and metastasis after surgery remains an important area of unmet clinical need.

Neoadjuvant virotherapy has the potential to aid eradication of in situ microscopic disease. Tai *et al* have demonstrated that a single intravenous dose of replicating pox virus administered prior to surgery could reverse surgical stress-induced NK cell suppression as well as the associated enhanced metastases in experimental models of metastatic breast cancer and melanoma.^6^ A similar result was also obtained with inactivated influenza vaccine^33^ and maraba virus,^34^ all mediated through enhanced NK cellular activity. More recently, the role of Maraba and other OV, although notably not VV, in control of postsurgical breast cancer recurrence has also been reported.^35 36^ These studies are encouraging, but as yet lack long-term survival data. Here, we demonstrate rational modification of the VV genome that promotes its efficacy as both a primary therapeutic agent via improved activation of adaptive immunity and as a neoadjuvant agent via improved activation of innate immunity required for postsurgical control of disease.

Most viruses have acquired strategies to ensure survival, replication and propagation in their hosts. These are mediated by different virulence gene products and in VV the N1L gene product has been shown to be vital for inhibition of innate immune responses against the virus.^37^ The N1L product has been demonstrated as a potent inhibitor of NF-κB signalling and thus inflammation required for T cell activation.^16^ We reasoned that deletion of the N1L gene from VV may serve to locally enhance the innate antiviral immune responses consequent to viral oncolysis and that i.t., as opposed to systemic, injection of the tumor bed prior to excision would allow for repeat dosing that provides a powerful mechanism by which to activate local immune responses required for prevention of postsurgical tumor outgrowth.

Using a panel of murine and human tumor cell lines, we found that VVL15∆N1L was at least as potent, if not more so than N1L-intact VV (VVΔTK) at killing murine-derived cancer cells in vitro, a finding that may not be surprising given the potential antiapoptotic function of the N1L protein.^38^

Here, we demonstrate that i.t.-administered VVΔTK∆N1L was able to reduce metastases of LLC tumors from the primary site. Possible explanations for this effect could include blockage of peritumor vasculature by enhanced leukocyte infiltration, enhanced numbers and activity of circulating NK cells and/or enhanced antitumor immune surveillance. i.t. neutrophil infiltration following virotherapy has previously been reported^27^ and VVΔTK∆N1L appeared to enhance this response. The prolonged presence of neutrophils within the TME has been associated with tumor progression via multiple mechanisms^39^ and it is interesting to note that KC, a murine neutrophil chemoattractant, was constitutively secreted by LLC tumor cells in vitro. Analogous to GMCSF, in the resting state, KC may act as an autocrine growth factor for some tumor cells. By contrast, recruitment and stimulation of neutrophils, as occurs following virotherapy, have also been associated with tumor cell cytotoxicity and suppression.^40^ The latter is attributed to mechanisms such the mechanical blockage of tumor vessels, excess release of cytotoxic agents, enhanced antigen-dependent cellular cytotoxicity in the presence of antitumor antibodies, or indeed through a phenotypic switch of neutrophils to being able to present scavenged tumor antigens^41^ and an in-depth analysis of the neutrophil phenotype induced by VVΔTK∆N1L is warranted to determine the extent to which neutrophil infiltration controls tumor progression.

A screen of inflammatory cytokines and chemokines within virally infected LLC flank tumors revealed the selective enhancement of KC, GCSF, IL1α/β and MIP1α expression. These are all regulated by NF-κB transcription factors and their expression might have been expected to be enhanced given the suppressive effect of the N1L protein on this pathway.^37^ Based on their well-characterized functions, enhanced production of KC (mainly from infected tumor cells) and GCSF was most likely to be responsible for the enhanced infiltration of i.t. neutrophils seen in vivo following VVΔTK∆N1L infection. These neutrophils, in turn, are likely a major source of IL1β and MIP1α in vivo, which display potent chemotactic activity for macrophages, dendritic, NK and T cells.^42^

VVΔTK∆N1L infection upregulates the expression of the IL1 family of cytokines from both DCs and macrophages: IL1α, IL1β and IL18. The latter two are surrogate markers for the activation of inflammasome platforms, a group of multimeric protein complexes that form in response to a range of exogenous and endogenously generated pathogen-associated or damage-associated molecules.^43^ Activated caspase-1 within the inflammasome is responsible for cleavage and activation of IL1β and IL18 precursor molecules. Although transcription of the precursor pro-IL1β gene is under the control of NF-κB transcription factors, pro-IL18 mRNA is constitutively expressed and has different regulatory controls.^43^ The fact that in our studies IL18 was also upregulated by VVL15∆N1L infection suggests that the N1L protein may additionally regulate inflammasome signaling by a previously undescribed mechanism of action. This warrants further investigation.

In comparison to VVΔTK, VVΔTK∆N1L infection enhanced the global activation of DCs and macrophages and in the latter also enhanced the upregulation of MHCII, effectively transforming them into professional APCs. It has been reported that human IL-8, the murine KC analogue, has the capacity to retain DCs in the tumor, preventing trafficking to lymphoid organs for antigen presentation.^44^ Here, we found that in vivo, i.t.-delivered VVL15∆N1L was able to enhance the percentage of DCs in murine spleens, but further analysis of their phenotype would be important for translation of this therapy. Given the effects of VVΔTK∆N1L on innate immune populations, particularly those considered important for controlling postsurgical metastasis, we postulated that VV∆TK∆N1L might be an effective neoadjuvant to conventional surgery for tumor treatment. In order to assess this, we analyzed different surgical models in the neoadjuvant setting. Tumors were excised 5–7 days after the final dose of virus to allow mice to recover from the effects of a protracted course of viral therapy and to provide adequate time for the development of an antitumor adaptive immune response. Importantly, our experiments have demonstrated that an elevated systemic NK response is sustained for at least 2 weeks following i.t. virus and therefore should last throughout the critical perioperative period. The efficacy results demonstrated a significant survival benefit for mice pretreated with VV∆TK∆N1L prior to surgical excision of their primary tumor. This effect was mediated by the enhanced elevation of NK cells, evidenced by the complete abrogation of response when the experiment was repeated in mice in which NK cells were depleted.

Preclinical and clinical data suggest that the efficacy of OV is significantly improved by arming the virus with an immunomodulatory gene, to encourage destruction of the immune-suppressive environment of the tumor and promote long-term antitumor surveillance. IL-12 has emerged as one of the most promising cytokines for antitumor therapeutics. Importantly, in the context of neoadjuvant therapy, it is a potent activator of both NK and T cell IFN-γ production,^45^ has been demonstrated to have antiangiogenic properties^46^ and can also upregulate cellular expression of MHCI and MHCII molecules,^47^ facilitating presentation of tumor antigens important for long-term disease surveillance. However, its use has been limited by the severe toxicity induced on systemic administration. Use in a neoadjuvant setting however, by i.t. administration prior to surgery, circumvents this toxicity and we have demonstrated that i.t. administration of IL-12 in the context of OV is a safe and effective way to enhance the effects of VV∆TK∆N1L as both a primary therapeutic agent and as a neoadjuvant agent. Indeed, neoadjuvant treatment with VV∆TK∆N1L-m/hIL12 completely prevented postoperative recurrence in vivo using head and neck cancer models that are intrinsically amenable to i.t. injection of virus, suggesting presurgical treatment with IL-12 armed VV can induce robust antitumor immune responses that significantly extend mortality. Interestingly, the power of immune checkpoint inhibitor (ICI) molecules as neoadjuvant agents with the puropse of inducing antitumor T cell reponses prior to surgical excision has been investigated clinically, with some pathological responses shown.^48^ OV such as TVEC and VV have been demonstrated to induce CD8 +T cell infiltration into tumors and upregulate expression of CTLA4, PD-L1 and other immune checkpoint molecules which would normally block T cell activation^35 36 49^ and as such can sensitize tumors to ICI,^50^ suggesting that neoadjuvant treatment with VV∆TK∆N1L-IL12 would have an additional advantage of ICI synergy, which warrants further preclinical evaluation.

Although these studies were carried out using surgical excision of flank tumors, representing a tumor and immune environment less complex than would occur in the clinical scenario, the principles demonstrated suggest that VV∆TK∆N1L-IL12 is a rationally engineered viral platform with particular efficacy as a neoadjuvant to oncological surgery, where its properties may minimize postoperative solid tumor metastasis and recurrence, significantly prolonging survival.
