# Correction: *A new oncolytic Vaccinia virus augments antitumor immune responses to prevent tumor recurrence and metastasis after surgery*

**DOI:** 10.1136/jitc-2019-000415corr1

**Published:** 2020-04-16

Ahmed J, Chard LS, Yuan M, *et al*. A new oncolytic Vaccinia virus augments antitumor immune responses to prevent tumor recurrence and metastasis after surgery. J ImmunoTher Cancer 2020;8:e000415. doi: 10.1136/jitc-2019-000415

In this article, the author Joel Schwartz was incorrectly linked to affiliation 3 twice and authors Nicholas Lemoine and Yaohe Wang should be linked to affiliation 2 in addition to affiliation 1. The correct author and affiliation list are shown below:

Jahangir Ahmed,^1^ Louisa S Chard,^1^ Ming Yuan,^1^ Jiwei Wang,^2^ Anwen Howells,^1^ Yuenan Li,^2^ Haoze Li,^2^ Zhongxian Zhang,^2^ Shuangshuang Lu,^2^ Dongling Gao,^2^ Pengju Wang,^2^ Yongchao Chu,^2^ Chadwan Al Yaghchi,^1^ Joel Schwartz,^3^ Ghassan Alusi,^1^ Nicholas Lemoine,^1,2^ Yaohe Wang^1,2^

^1^Centre for Biomarkers & Biotherapeutics, Barts Cancer Institute, Queen Mary University of London, London, UK

^2^National Centre for International Research in Cell and Gene Therapy, Zhengzhou University, Zhengzhou, Henan, China

^3^University of Illinois at Chicago, Chicago, Illinois, USA

