# Supplementary figures and images for: A new oncolytic Vaccinia virus augments antitumor immune responses to prevent tumor recurrence and metastasis after surgery
Source: J Immunother Cancer. 2020 Mar 26;8(1):e000415. doi: 10.1136/jitc-2019-000415 (PMC7206973; doi:10.1136/jitc-2019-000415)

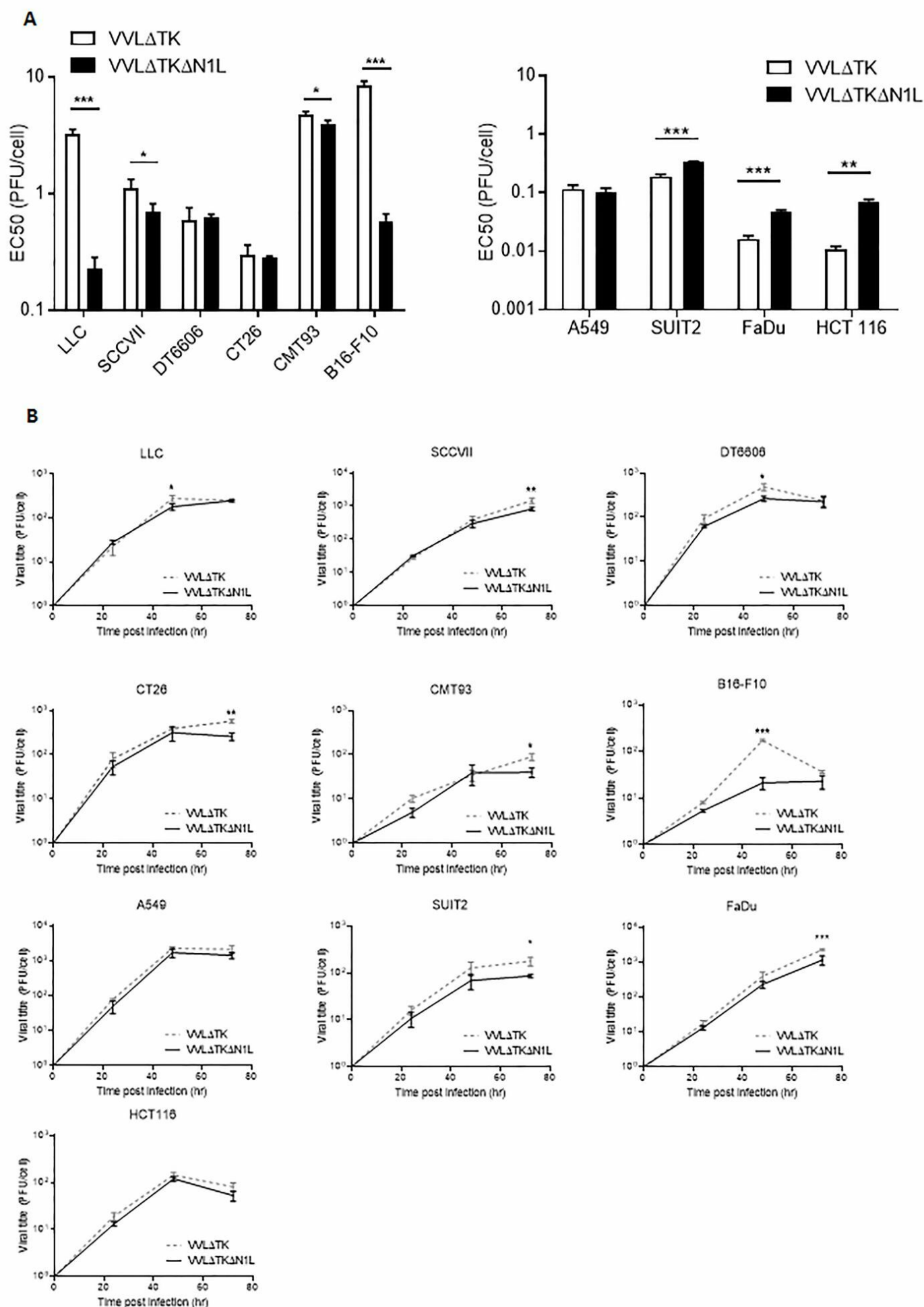

Supplement: Supplementary data [file jitc-2019-000415supp001.pdf]

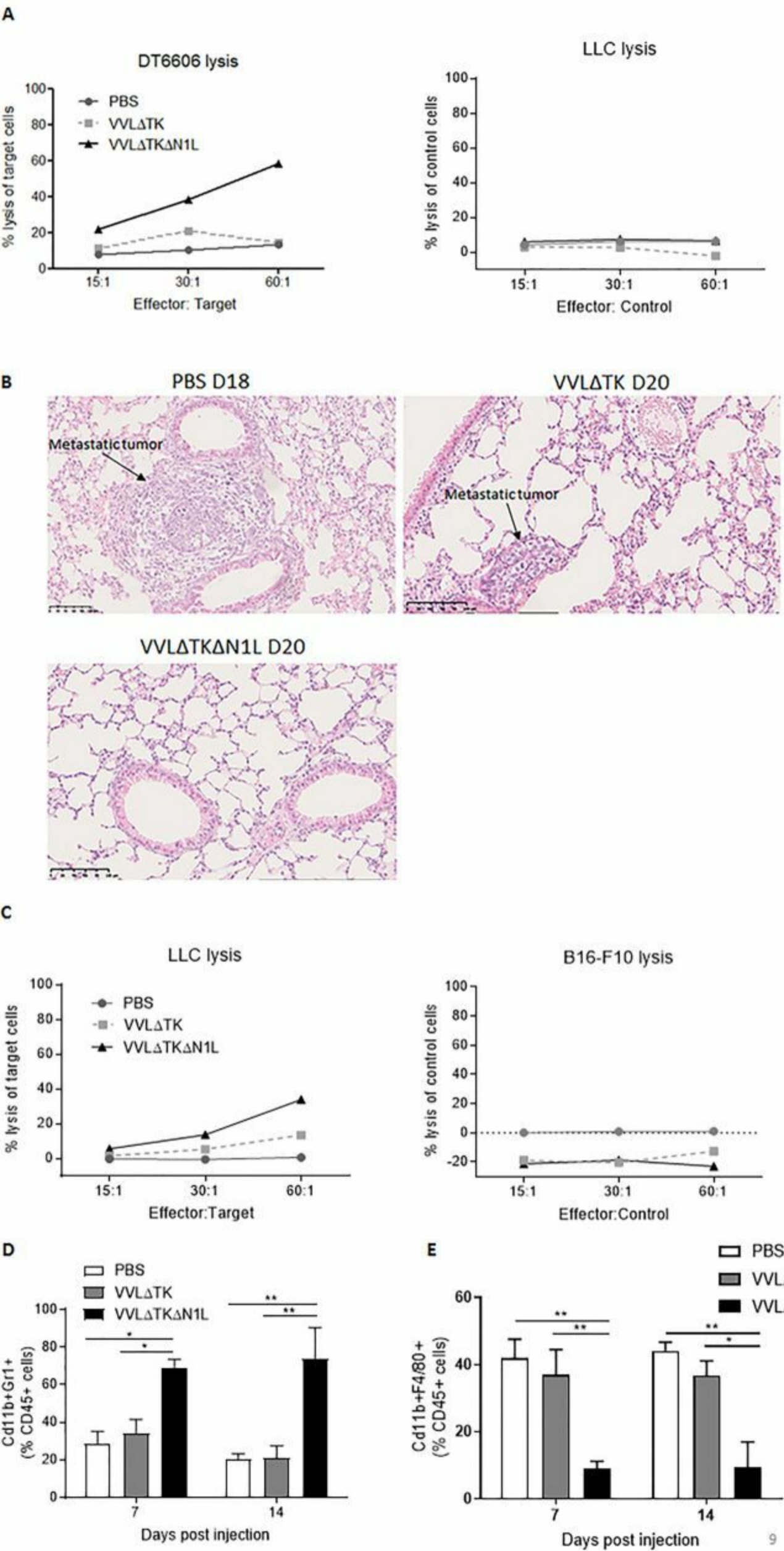

Supplement: Supplementary data [file jitc-2019-000415supp002.pdf]

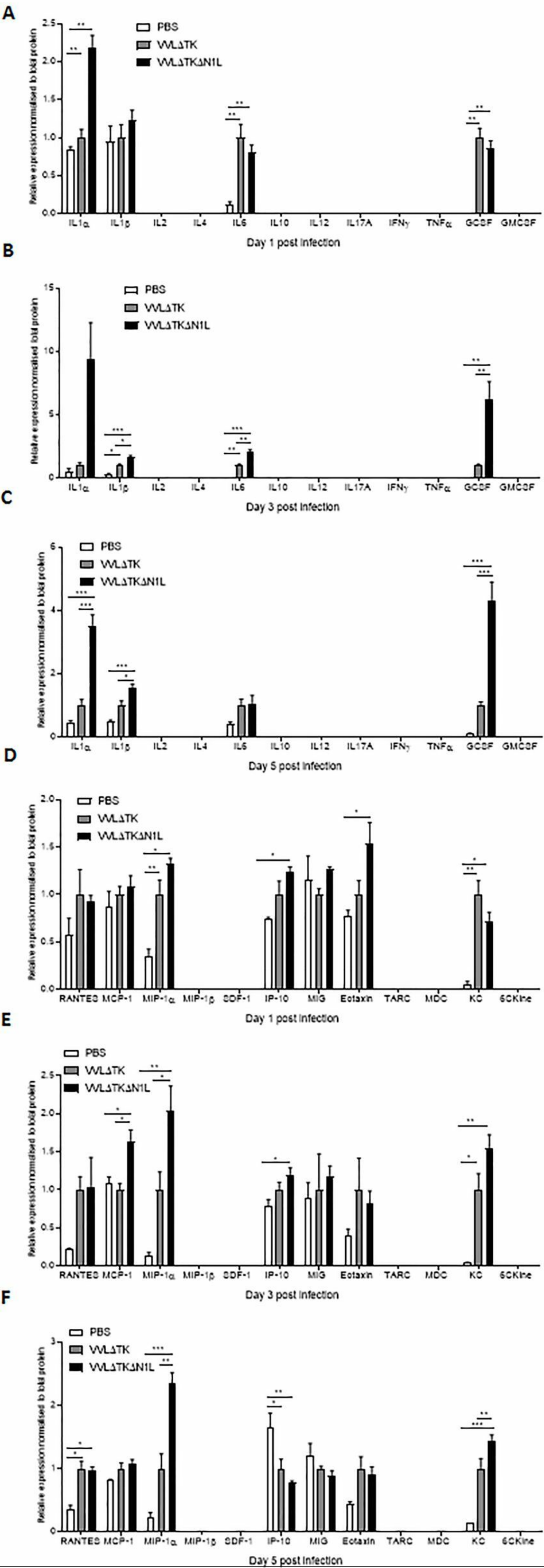

Supplement: Supplementary data [file jitc-2019-000415supp003.pdf]

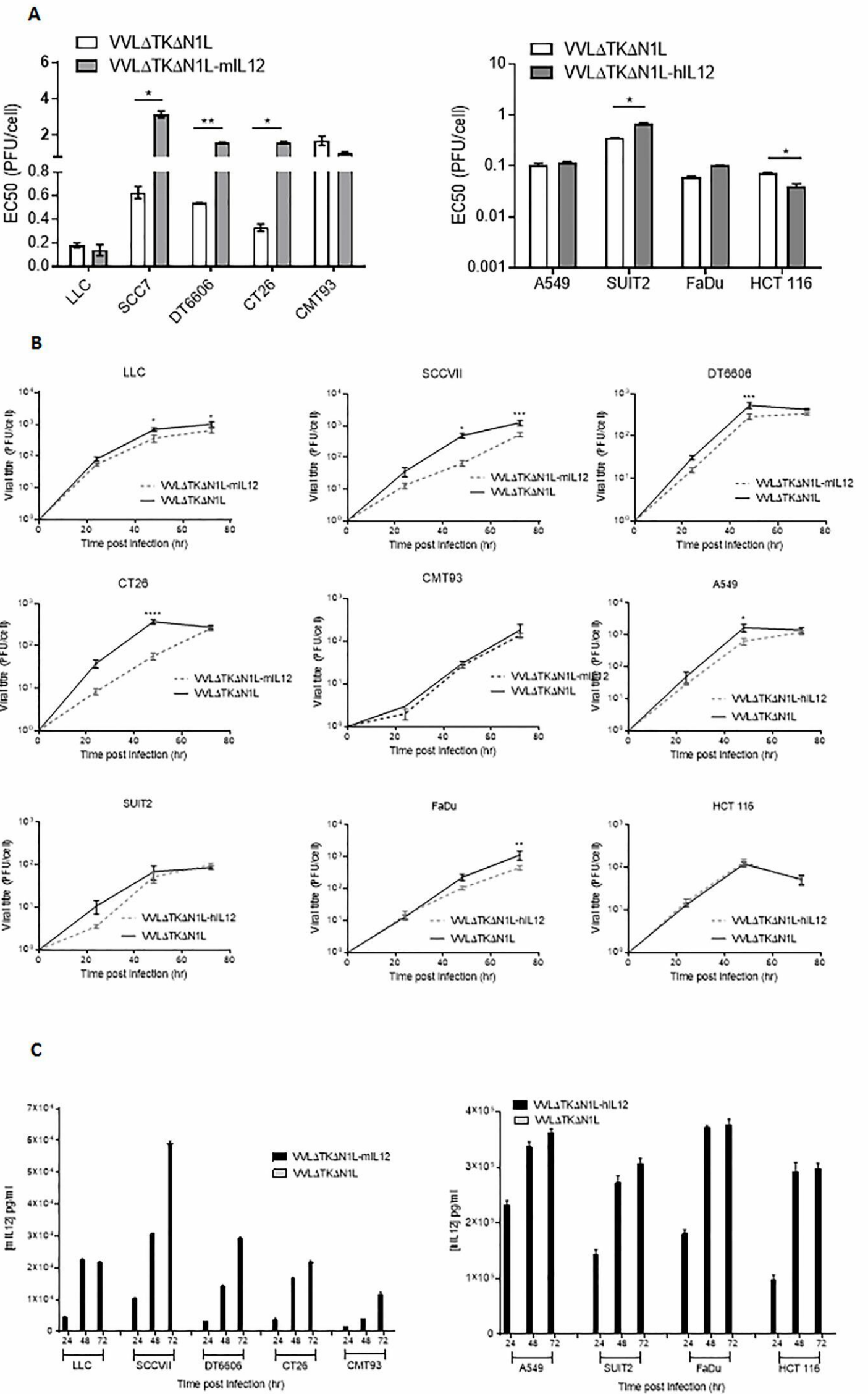

Supplement: Supplementary data [file jitc-2019-000415supp004.pdf]

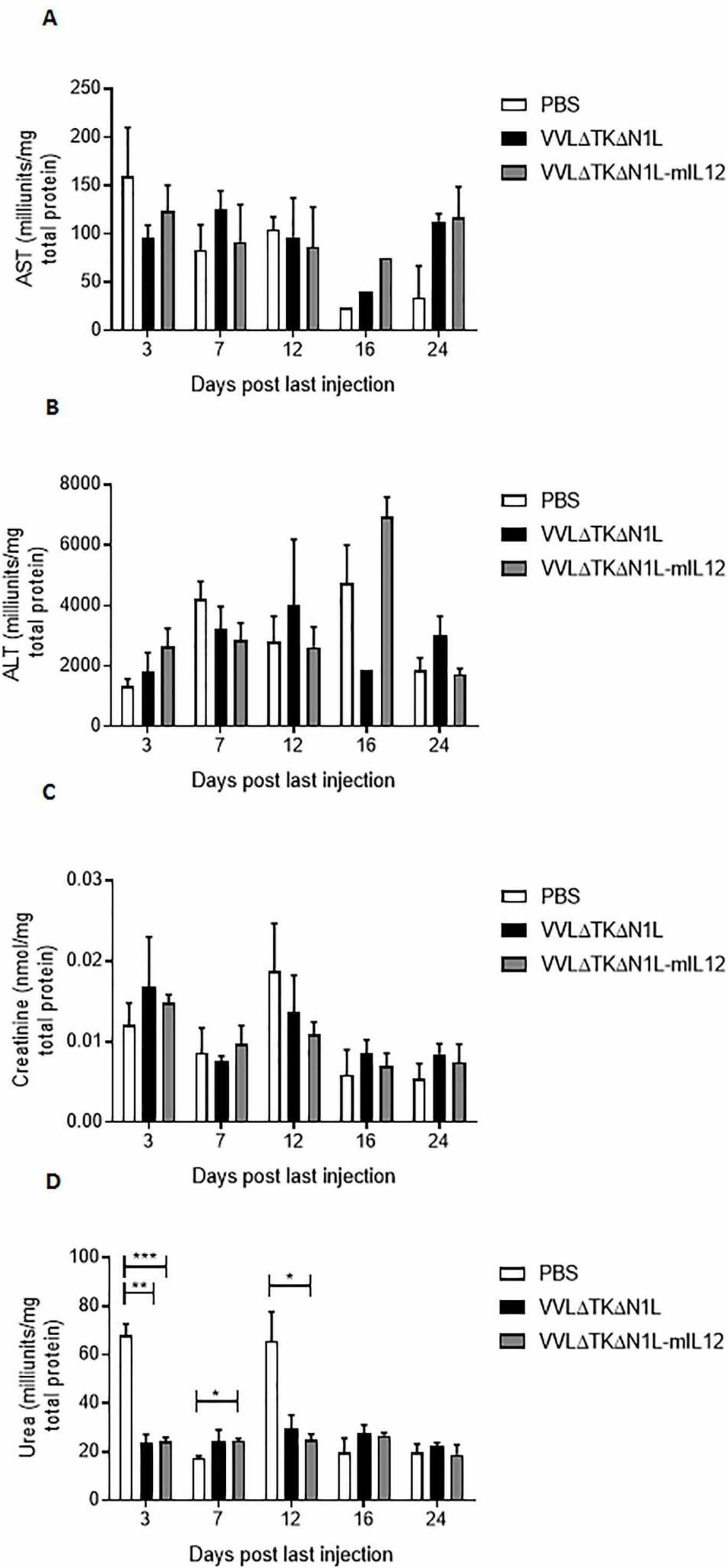

Supplement: Supplementary data [file jitc-2019-000415supp005.pdf]
