# Supplementary material for: A new oncolytic Vaccinia virus augments antitumor immune responses to prevent tumor recurrence and metastasis after surgery
Source: J Immunother Cancer. 2020 Mar 26;8(1):e000415. doi: 10.1136/jitc-2019-000415 (PMC7206973; doi:10.1136/jitc-2019-000415)
Supplement: Supplementary data [file jitc-2019-000415supp006.pdf]

A new oncolytic Vaccinia virus augments anti-tumor immune responses to prevent tumor recurrence and metastasis after surgery

Running Title: Oncolytic Vaccinia virus as a neoadjuvant agent

Jahangir Ahmed[1*], Louisa S Chard[1*], Ming Yuan[1*], Jiwei Wang[2], Anwen Howells[1], Yuenan Li[2], Haoze Li[2], Zhongxian Zhang[2], Shuangshuang Lu[2], Dongling Gao[2], Pengju Wang[2], Chadwan Al Yaghchi[1],Joel Schwartz[3], Ghassan Alusi[1], Nick Lemoine[1,2], Yaohe Wang[1,2]

Supplementary Figure Legends

Supplementary Figure 1. VVΔTKΔN1L replicates in and kills murine and human tumor cell lines. (**A**) Cytotoxicity of VVLΔTK and VVΔTKΔN1L against murine Lewis lung cancer (LLC), murine head and neck cancer SCCVII, murine pancreatic cancer DT6606, murine colon carcinoma CT26, murine colon carcinoma CMT93 and murine breast cancer B16-F10 (top graph) and human lung cancer A549, human pancreatic cancer (SUIT2), human pharyngeal carcinoma FaDu and human colon carcinoma HCT-116 (bottom graph). Cell death was determined by MTS assay 144 hours post-infection. Mean EC50 values ± SEM are shown. A paired students T test was used to assess significance. (**B**) Production of infectious virions after infection of murine and human tumor cell lines at 1 PFU/cell for 24-96 hours. Mean viral replication ± SEM was determined by TCID50 assay on CV1 cells. Statistical analysis was carried out using a two-way ANOVA with post hoc Tukey tests. In all cases, the mean ± SEM is shown. *$p<0.05$; **$p<0.01$; ***$p<0.001$; ****$p<0.0001$.

Supplementary Figure 2. VVΔTKΔN1L enhances intra-tumoral neutrophil and macrophage infiltration. (A) DT6606 or LLC tumors were established in the flanks of immune-competent C57/Bl6 mice (n=7/group). Once palpable, mice were injected intra-tumorally (i.t) daily for 5 days with $1x10^8$PFU VVΔTK, VVΔTKΔN1L or PBS. Splenocytes from DT6606 (**A**) or LLC tumor-bearing animals were collected at day 14 and co-cultured with growth arrested tumor cells. 5 days later, splenocytes from each treatment group were pooled and co-cultured with 5000 target or control tumor cells at 15:1, 30:1 and 60:1 ratios. Non-radioactive LDH release assays were performed to determine percentage tumor cell lysis. (**B**) Lungs from LLC tumor bearing animals were collected at different time points after treatment with PBS, VVΔTK, VVΔTKΔN1L viruses ($1x10^8$pfu/injection) at 18-20 days post treatment, representative lung histopathology of H&E staining are presented. **(C-D)** DT6606 tumors were established in the flanks of immune-competent C57/Bl6 mice (n=3-4/group). Once palpable, mice were injected i.t once with $1x10^8$PFU VVΔTK, VVΔTKΔN1L or PBS. (**C**) FACS analysis was used to assess CD11b+Gr1+ neutrophils in tumors at the indicated time points. (**D**) FACS analysis was used to assess CD11b+F4/80+ macrophages in the tumors at the indicated time points. A one-way ANOVA with post hoc Tukey tests were used to assess significance at each time point. In all cases, the mean ± SEM is shown. *$p<0.05$; **$p<0.01$.

Ahmed J, *et al. J Immunother Cancer* 2020; 8:e000415. doi: 10.1136/jitc-2019-000415

Supplementary Figure 3. VVΔTKΔN1L alters *in vivo* secretion of immune modulators. LLC tumors were established in the flanks of immune-competent C57/Bl6 mice (n=3-4/group). Once palpable, mice were injected i.t once with 1x10^8PFU VVΔTK, VVΔTKΔN1L or PBS. One-way ANOVA with post hoc Tukey tests was used to assess significance. (**A-C**) At 1, 3 and 5 days post-infection, tumors were harvested and homogenised and their supernatant analysed for the presence of 12 common inflammatory cytokines by multi analyte ELISA. Relative expression was normalised to total protein. (**D-F**) At 1, 3 and 5 days post-infection, tumors were harvested and homogenised and their supernatant analysed for the presence of 12 common inflammatory chemokines by multi analyte ELISA. Relative expression was normalised to total protein. A one-way ANOVA with post hoc Tukey tests were used to assess significance. In all cases, the mean ± SEM is shown. *p<0.05; **p<0.01; ***p<0.001; ****p<0.0001.

Supplementary Figure 4. VVΔTKΔN1L-IL12 replicates in and kills murine and human tumor cell lines. (**A**) Cytotoxicity of VVΔTKΔN1L and VVΔTKΔN1L-m/hIL12 against murine Lewis lung cancer (LLC), murine head and neck cancer SCCVII, murine pancreatic cancer DT6606, murine colon carcinoma CT26 and murine colon carcinoma CMT93 (top graph) and human lung cancer A549, human pancreatic cancer (SUIT2), human pharyngeal carcinoma FaDu and human colon carcinoma HCT-116 (bottom graph). Cell death was determined by MTS assay 144 hours post-infection. Mean EC50 values ± SEM are shown. A paired students T test was used to assess significance. (**B**) Production of infectious virions after infection of murine and human tumor cell lines at 1 PFU/cell for 24-96 hours. Mean viral replication ± SEM was determined by TCID50 assay on CV1 cells. Statistical analysis was carried out using a two-way ANOVA with post hoc Tukey tests. *p<0.5; **p<0.1; ***p<0.01. (**C**) IL-12 expression after infection of murine and tumor cell lines (1 PFU/cell) was determined at 24, 48 and 72 hours post infection using ELISA. In all cases, the mean ± SEM is shown. *p<0.05; **p<0.01; ***p<0.001; ****p<0.0001.

Supplementary Figure 5. VVΔTKΔN1L-IL12 does not perturb kidney and liver biochemistry after i.t administration into murine LY2 flank tumors. LY2 tumors were established and treated i.t with 1x10^8PFU VVΔTKΔN1L, VVΔTKΔN1L-mIL12 or PBS daily for 5 days (n=3/group). 3-24 days post last treatment, livers and kidneys were collected, homogenised and assessed for expression of aspartate aminotransferase (AST) (**A**), alanine aminotransferase (ALT) (**B**) in the liver samples, and Creatinine (**C**) and Urea (**D**) in the kidney samples using colorimetric assays as described in the methods. A one-way ANOVA with post hoc Tukey tests were used to assess significance at each time point. . In all cases, the mean ± SEM is shown. *p<0.05; **p<0.01; ***p<0.001; ****p<0.0001.

Ahmed J, *et al. J Immunother Cancer* 2020; 8:e000415. doi: 10.1136/jitc-2019-000415
